# Clinical Correlates and Prognostic Value of Low QRS Voltages in Non-Ischemic Dilated Cardiomyopathy: A Retrospective Cohort Study

**DOI:** 10.31083/RCM50413

**Published:** 2026-10-22

**Authors:** Tao Li, Tingting Tian, Xiaoxue Zhang, Zhichun Gao, Lijiao Zhang, Siming Gong, Xin Deng, Moyan Qin, Yuxiao Zhang, Zhexue Qin, Xiaolin Luo

**Affiliations:** ^1^Department of Cardiology, Xinqiao Hospital, Army Medical University (Third Military Medical University), 400037 Chongqing, China; ^2^Department of Emergency, Xinqiao Hospital, Army Medical University (Third Military Medical University), 400037 Chongqing, China; ^3^Department of Ultrasound, Xinqiao Hospital, Army Medical University (Third Military Medical University), 400037 Chongqing, China; ^4^Department of Geriatrics, Southwest Hospital, Army Medical University (Third Military Medical University), 400038 Chongqing, China

**Keywords:** electrocardiography, dilated cardiomyopathy, prognosis, heart failure

## Abstract

**Background:**

Data on the prognostic significance of low QRS voltage (LQRSV) in non-ischemic dilated cardiomyopathy (NIDCM) are limited. Therefore, this study aimed to determine whether LQRSV is associated with a composite endpoint of all-cause mortality or heart failure (HF) re-hospitalization in patients with NIDCM.

**Methods:**

Patients with NIDCM who met predefined criteria were retrospectively enrolled and divided into LQRSV and non-LQRSV groups based on electrocardiographic findings. The primary outcome was a composite of all-cause mortality or HF re-hospitalization. Secondary outcomes included all-cause mortality, cardiovascular (CV) death, and HF re-hospitalization. The prognostic significance of LQRSV was evaluated using Kaplan–Meier analysis and Cox proportional hazards models. Restricted cubic spline (RCS) functions were applied to examine potential nonlinear relationships between continuous QRS voltage and the primary outcome. Receiver operating characteristic (ROC) curves were used to assess predictive performance. Propensity score matching (PSM) was performed to balance baseline characteristics, and sensitivity and subgroup analyses were conducted to test the robustness of the findings.

**Results:**

The overall cohort included 784 consecutive patients with NIDCM (median age 56 years; 30% female). During a median follow-up of 1079 days, 335 primary outcome events occurred. After PSM, 463 patients were matched, with 186 in the LQRSV group and 277 in the non-LQRSV group. In the matched cohort, Kaplan–Meier analysis with the log-rank test showed that the LQRSV group had a significantly worse long-term prognosis than the non-LQRSV group (*p* < 0.001). Multivariable Cox analyses demonstrated that LQRSV was significantly associated with the composite of all-cause mortality or HF re-hospitalization (hazard ratio, (HR) 2.048, 95% confidence interval (CI) 1.519–2.760; *p* < 0.001), all-cause mortality (HR 2.526, 95% CI 1.660–3.844; *p* < 0.001), and CV death (HR 2.820, 95% CI 1.776–4.476; *p* < 0.001). RCS analyses showed a linear association (*p* for nonlinearity > 0.05), with the highest risk observed at the lowest QRS voltage amplitudes. ROC analysis revealed that incorporating LQRSV into the clinical model significantly increased the area under the curve (0.808 vs. 0.723; *p* < 0.001).

**Conclusions:**

LQRSV is associated with poor prognosis in NIDCM. Routine electrocardiographic assessment of QRS voltage may provide valuable information for risk stratification.

## 1. Introduction

Non-ischemic dilated cardiomyopathy (NIDCM) is a heterogeneous myocardial disease characterized by left ventricular (LV) dilation and dysfunction, often leading to heart failure (HF) and death [1,2]. Although the long-term prognosis of NIDCM has improved because of advances in understanding pathophysiology and treatment strategies, a substantial incidence of HF and mortality persists [3,4]. Therefore, identifying patients at risk of death or progressive deterioration requiring hospitalization remains critical.

Electrocardiography (ECG) is a reproducible, non-invasive, low-cost, and widely used clinical diagnostic and prognostic tool. Previous studies have demonstrated the prognostic value of ECG in NIDCM [5,6,7]. Low QRS voltage (LQRSV), an uncommon ECG pattern, has been observed in a wide range of populations, including those with cardiovascular (CV) diseases, those with non-CV diseases, and even healthy individuals [8,9,10,11,12,13]. However, in the context of CV disease, prior studies found that LQRSV most commonly occurs in diseases such as cardiac amyloidosis, non-ischemic LV scar, or arrhythmogenic cardiomyopathy [14,15,16]. In patients with NIDCM, more commonly observed ECG patterns, such as left bundle branch block (LBBB) and atrial fibrillation (AF), have received greater attention as prognostic markers [17]. LQRSV has been reported in some patients with NIDCM, likely reflecting extensive myocardial cell loss and fibrosis [5]. However, the prognostic impact of LQRSV in NIDCM remains poorly studied, and its associated risks and prognostic significance remain unclear. We aimed to explore the possible long-term prognostic value of LQRSV in patients with NIDCM.

## 2. Materials and Methods

### 2.1 Study Design and Population

This retrospective study enrolled patients with NIDCM between December 2014 and May 2024 at Xinqiao Hospital, Army Medical University, a tertiary teaching hospital in Southwest China, providing care for both local patients and patients referred from a broad region of Southwest China. This study was approved by the Ethics Committee of Xinqiao Hospital (No. 2023-089-01) and adhered to the Declaration of Helsinki. Owing to its retrospective nature involving a review of medical records, the committee issued a waiver of informed consent.

Patients with a confirmed diagnosis of NIDCM were included if baseline echocardiographic and ECG data were available. NIDCM was defined according to established clinical and imaging criteria [18], characterized by LV dilatation and systolic dysfunction unexplained solely by abnormal loading conditions or coronary artery disease [19,20]. Patients with ischemic cardiomyopathy or significant coronary artery disease, congenital heart disease, and significant valvular heart disease were excluded. Standard 12-lead ECGs were recorded in the supine position with limb electrodes placed at the wrists and ankles, using a paper speed of 25 mm/s and a voltage calibration of 10 mm/mV. The baseline ECG was defined as the standard 12-lead ECG obtained at the patient’s first diagnosis of NIDCM. LQRSV was defined as QRS amplitude <0.5 mV in all limb leads and/or <1.0 mV in all precordial leads [9,21]. Inclusion criteria were (i) age ≥18 years; (ii) complete baseline data; and (iii) no history of other diseases that may cause LQRSV, including pericardial effusion, pleural effusion, chronic obstructive pulmonary disease (COPD), or infiltrative cardiomyopathy such as cardiac amyloidosis. Patients with NIDCM were classified into LQRSV or non-LQRSV groups.

### 2.2 Data Collection

A systematic evaluation of patients with NIDCM was performed by reviewing medical records, relevant laboratory and imaging findings, and therapeutic management in compliance with current guidelines [19]. Baseline data were collected during enrollment, including demographics, clinical history, laboratory examination, echocardiographic results, ECG parameters, and medications. Demographic information included age, sex, body mass index (BMI), smoking and drinking status, systolic blood pressure (SBP), diastolic blood pressure (DBP), heart rate, and New York Heart Association (NYHA) cardiac function status. Clinical history included comorbidities such as hypertension, diabetes mellitus (DM), chronic kidney disease (CKD), and hepatic insufficiency. Laboratory tests included estimated glomerular filtration rate (eGFR), serum creatinine, B-type natriuretic peptide (BNP), and N-terminal pro B-type natriuretic peptide (NT-proBNP). ECG parameters included QRS duration and voltage, AF, LBBB, nonsustained ventricular tachycardia (NSVT), intraventricular block (IVB), fragmented QRS complex (fQRS), left anterior fascicular block (LAFB), left posterior fascicular block (LPFB), first-degree atrioventricular block (AVB), and second-degree AVB. Echocardiographic parameters included left ventricular end-diastolic diameter index (LVEDDI), left ventricular ejection fraction (LVEF), mitral regurgitation (MR), tricuspid regurgitation (TR), and aortic regurgitation (AR). Medication therapy included β-blockers, sodium-glucose cotransporter 2 inhibitor (SGLT2i), angiotensin-converting enzyme inhibitors (ACEI), angiotensin receptor blockers (ARB), angiotensin receptor neprilysin inhibitors (ARNI), mineralocorticoid receptor antagonists (MRA), diuretics, and digoxin. Data on device therapy included implantable cardioverter defibrillator (ICD) and cardiac resynchronization therapy (CRT). BNP levels were converted to NT-proBNP using the following formula: log NT-proBNP = 1.21 + 1.03 × log BNP − 0.009 × BMI − 0.007 × eGFR [22].

### 2.3 Study Outcomes and Follow-up

The primary outcome was a composite of all-cause mortality or HF re-hospitalization. Secondary outcomes were all-cause mortality, CV death, and HF re-hospitalization during follow-up. CV death was defined as death attributable to a defined cardiovascular cause, including sudden cardiac death, pump failure death due to HF, arrhythmia or conduction disturbance, thromboembolism, stroke, or other clearly documented cardiovascular causes [23]. HF re-hospitalization was defined as an unplanned hospital admission requiring an overnight stay or a hospital stay of at least 24 hours, with worsening HF as the primary reason for admission, accompanied by new or worsening HF symptoms/signs and initiation or intensification of HF-specific therapy [23]. Patients were followed up by experienced physicians through medical records, and the follow-up was terminated on July 31, 2025.

### 2.4 Statistical Analysis

Continuous variables are presented as medians (interquartile range, IQR), and categorical variables as frequencies (percentages). The Mann–Whitney U test or χ^2^ test was used to compare continuous and categorical variables, respectively, between groups.

Event-free survival related to the study outcomes was estimated using Kaplan–Meier analysis, and survival curves were compared using the log-rank test. Candidate predictors for the study outcomes were evaluated utilizing univariable Cox proportional hazards models. Multicollinearity among covariates was assessed using the variance inflation factor (VIF) for each variable in the model. Variables with VIF >3 were considered potentially problematic. All variables with *p* < 0.05, after excluding collinear predictors based on VIF, were included in the final multivariable Cox proportional hazards model via stepwise bootstrap. The area under the receiver operating characteristic (ROC) curve (AUC) was then used to evaluate the association between LQRSV and the composite outcome of all-cause mortality or HF re-hospitalization. The difference in AUC between Model 1 and Model 2 was assessed using the DeLong test.

Three-knots restricted cubic spline (RCS) plots were employed to explore potential inflection points and assess the linear or nonlinear relationships between clinical outcomes and QRS voltage in limb leads and the SV1 + RV5 voltage as continuous variables.

To reduce the impact of potential confounders, propensity score matching (PSM) was performed to balance confounding factors between patients with and without LQRSV. The propensity score was estimated using logistic regression, incorporating the following covariates: age, sex, BMI, smoking and drinking status, NYHA class III/IV, SBP, hypertension, CKD, hepatic insufficiency, AF, LBBB, QRS duration, LVEDDI, LVEF, log NT-proBNP, ICD, and CRT. Patients with LQRSV were matched with non-LQRSV controls in a 1:2 ratio using nearest-neighbor matching based on propensity scores, with a caliper width of 0.1 [24]. Covariate balance post-matching was assessed using standardized mean differences, with values <0.1 considered indicative of adequate balance [25].

A subgroup analysis of the primary outcome was conducted according to the two study groups based on age, sex, BMI, smoking, drinking, SBP, heart rate, hypertension, DM, CKD, hepatic insufficiency, AF, LBBB, IVB, NSVT, NYHA class III/IV, LVEDDI, LVEF, moderate-to-severe MR, moderate-to-severe AR, moderate-to-severe TR, ICD, and CRT. Results were expressed as hazard ratios (HR) and 95% confidence intervals (CI). Sensitivity analyses were performed for the overall cohort to test the robustness of the findings. Multivariable Cox proportional hazards models were used to analyze the effects of covariates on the composite outcome of all-cause mortality or HF re-hospitalization, as well as all-cause mortality, CV death, and HF re-hospitalization.

All statistical analyses were conducted using R software (version 4.4.3; R Foundation for Statistical Computing, Vienna, Austria). Two-sided *p*-values < 0.05 were considered statistically significant.

## 3. Results

### 3.1 Baseline Characteristics

A total of 784 patients with NIDCM were included in the overall cohort: 217 in the LQRSV group and 567 in the non-LQRSV group. After PSM, 463 patients were matched, with 186 and 277 patients in the LQRSV and non-LQRSV groups, respectively (Fig. 1).

**Fig. 1.**
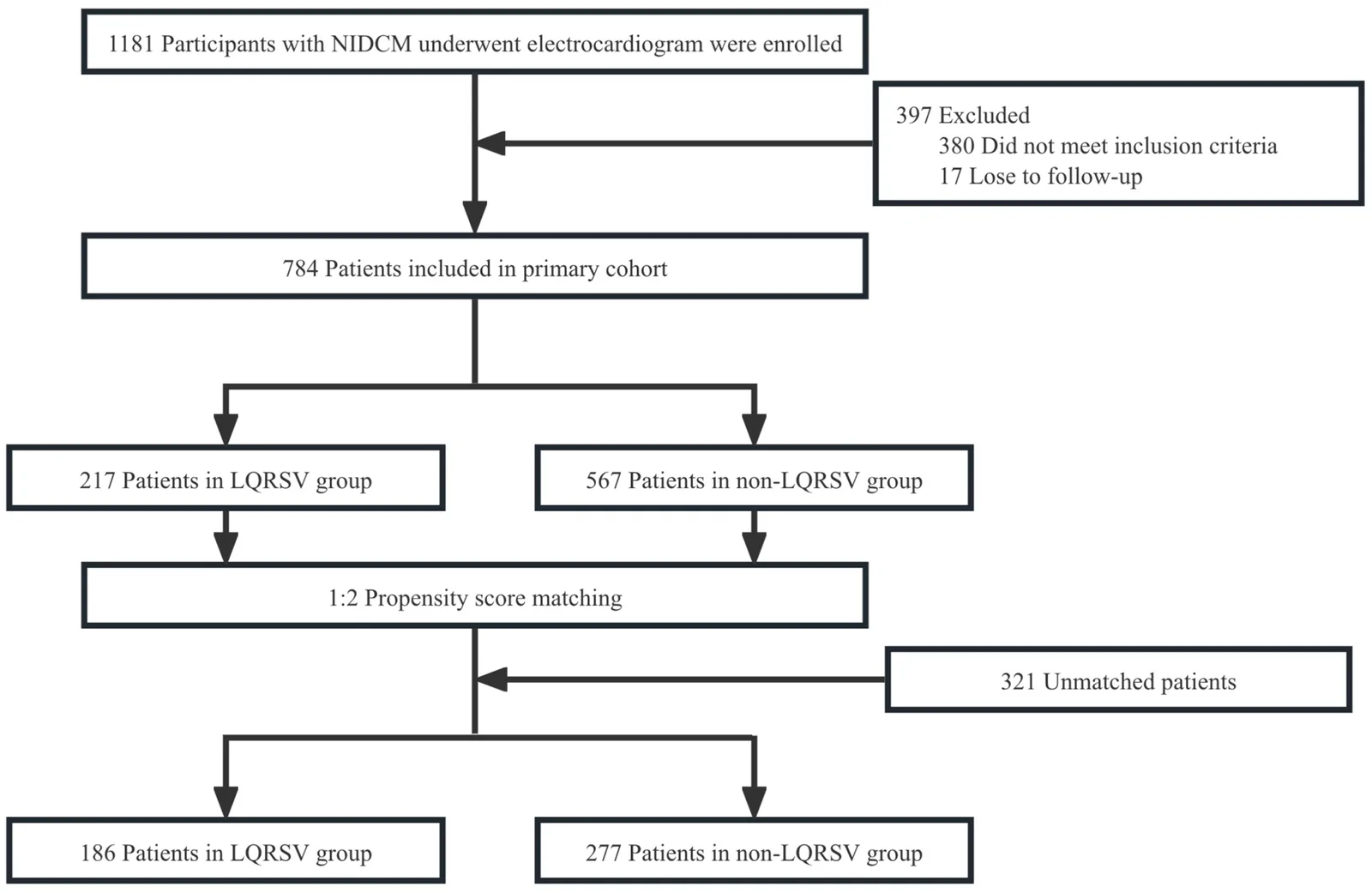
**Study flow chart of enrollment**. LQRSV, low QRS voltages; NIDCM, non-ischemic dilated cardiomyopathy.

Baseline characteristics before and after PSM are presented in Table 1. Before matching, the LQRSV group had a lower median BMI, SBP, QRS duration, and eGFR levels, and a higher proportion of patients with NYHA class III/IV, CKD, and hepatic insufficiency compared with the non-LQRSV group. The LQRSV group also showed higher prevalence of AF, fQRS, moderate-to-severe MR, moderate-to-severe TR, and elevated NT-proBNP and serum creatinine. Regarding pharmacotherapeutic and device-based interventions, the LQRSV group had lower utilization rates of β-blockers, SGLT2i, ACEI/ARB/ARNI, and CRT, but higher prevalence of loop diuretics, digoxin, and ICD. PSM substantially improved covariate balance between the groups (**Supplementary Table 1**). After matching, most differences were attenuated, except for a higher prevalence of fQRS, moderate-to-severe MR and TR, and lower use of β-blockers, SGLT2i, and ACEI/ARB/ARNI in the LQRSV group.

**Table 1.** **Baseline characteristics in patients with NIDCM**.

		Before PSM			After PSM		
		Non-LQRSV (n = 567)	LQRSV (n = 217)	*p*-value	Non-LQRSV (n = 277)	LQRSV (n = 186)	*p*-value
Age, years, median (IQR)		57 (48, 64)	54 (44, 67)	0.399	57 (49, 66)	56 (45, 68)	0.596
Male, n (%)		400 (70.55)	149 (68.66)	0.607	193 (69.68)	130 (69.89)	0.960
BMI, kg/m^2^, median (IQR)		24.2 (21.5, 27.0)	23.3 (20.4, 26.0)	0.005	23.6 (21.1, 27.0)	23.3 (20.5, 26.4)	0.391
Smoking, n (%)		247 (43.56)	98 (45.16)	0.687	124 (44.77)	82 (44.09)	0.885
Drinking, n (%)		153 (26.98)	49 (22.58)	0.207	67 (24.19)	46 (24.73)	0.894
NYHA class III/IV, n (%)		272 (47.97)	173 (79.72)	<0.001	192 (69.31)	142 (76.34)	0.098
SBP, mmHg, median (IQR)		113 (100, 126)	103 (91, 117)	<0.001	107 (95, 122)	106 (95, 120)	0.519
DBP, mmHg, median (IQR)		71 (62, 80)	69 (60, 78)	0.100	70 (61, 79)	70 (62, 80)	0.557
Heart rate, beats/min, median (IQR)		83 (71, 99)	86 (74, 102)	0.127	87 (71, 106)	86 (72, 101)	0.428
Hypertension, n (%)		155 (27.34)	38 (17.51)	0.004	59 (21.30)	36 (19.35)	0.611
DM, n (%)		107 (18.87)	45 (20.74)	0.554	52 (18.77)	40 (21.51)	0.470
CKD, n (%)		65 (11.46)	46 (21.20)	<0.001	47 (16.97)	33 (17.74)	0.829
Hepatic insufficiency, n (%)		32 (5.64)	33 (15.21)	<0.001	27 (9.75)	23 (12.37)	0.374
QRS duration, ms, median (IQR)		104 (90, 132)	98 (86, 116)	<0.001	100 (86, 124)	98 (86, 118)	0.510
fQRS, n (%)		109 (19.22)	78 (35.94)	<0.001	57 (20.58)	65 (34.95)	<0.001
AF, n (%)		86 (15.17)	62 (28.57)	<0.001	60 (21.66)	53 (28.49)	0.093
IVB, n (%)		49 (8.64)	24 (11.06)	0.297	33 (11.91)	23 (12.37)	0.884
LBBB, n (%)		108 (19.05)	17 (7.83)	<0.001	29 (10.47)	17 (9.14)	0.639
NSVT, n (%)		13 (2.29)	7 (3.23)	0.458	7 (2.53)	6 (3.23)	0.655
LPFB, n (%)		6 (1.06)	2 (0.92)	0.999	2 (0.72)	1 (0.54)	1.000
LAFB, n (%)		26 (4.59)	16 (7.37)	0.121	13 (4.69)	15 (8.06)	0.136
First-degree AVB, n (%)		44 (7.76)	12 (5.53)	0.278	21 (7.58)	10 (5.38)	0.352
Second-degree AVB, n (%)		11 (1.94)	5 (2.30)	0.779	8 (2.89)	5 (2.69)	0.898
Minimum limb-lead voltage, mV, median (IQR)		0.40 (0.30, 0.50)	0.11 (0.10, 0.20)	<0.001	0.40 (0.30, 0.50)	0.11 (0.10, 0.20)	<0.001
Voltage sum in limb leads, mV, median (IQR)		4.21 (3.35, 5.35)	1.40 (1.08, 1.72)	<0.001	4.04 (3.15, 5.10)	1.40 (1.08, 1.72)	<0.001
SV1 + RV5 voltage, mV, median (IQR)		2.86 (1.96, 3.84)	1.56 (1.04, 2.19)	<0.001	2.76 (1.86, 3.71)	1.61 (1.10, 2.20)	<0.001
LVEDDI, mm/m^2^, median (IQR)		45 (38, 54)	47 (40, 56)	0.106	46 (39, 55)	47 (40, 56)	0.646
LVEF, median (IQR)		36 (28, 44)	35 (29, 42)	0.343	34 (28, 41)	35 (29, 42)	0.870
MR (moderate/severe), n (%)		312 (55.03)	152 (70.05)	<0.001	167 (60.29)	131 (70.43)	0.026
AR (moderate/severe), n (%)		43 (7.58)	11 (5.07)	0.214	20 (7.22)	9 (4.84)	0.300
TR (moderate/severe), n (%)		156 (27.51)	127 (58.53)	<0.001	104 (37.55)	108 (58.06)	<0.001
Log NT-proBNP, median (IQR)		3.12 (2.56, 3.61)	3.51 (3.21, 3.97)	<0.001	3.37 (2.83, 3.86)	3.44 (3.18, 3.83)	0.144
Serum creatinine, μmol/L, median (IQR)		82 (69, 97)	88 (74, 111)	<0.001	85 (70, 103)	88 (74, 108)	0.303
eGFR, mL/min/1.73 m^2^, median (IQR)		85 (71, 99)	76 (59, 95)	<0.001	81 (65, 96)	77 (62, 97)	0.592
Medication							
	β-blockers, n (%)	538 (94.89)	189 (87.10)	<0.001	260 (93.86)	162 (87.10)	0.012
	SGLT2i, n (%)	436 (76.90)	47 (21.66)	<0.001	210 (75.81)	39 (20.97)	<0.001
	ACEI/ARB/ARNI, n (%)	550 (97.00)	191 (88.02)	<0.001	265 (95.67)	169 (90.86)	0.036
	MRA, n (%)	473 (83.42)	183 (84.33)	0.758	245 (88.45)	157 (84.41)	0.208
	Loop diuretics, n (%)	193 (34.04)	116 (53.46)	<0.001	116 (41.88)	93 (50.00)	0.085
	Digoxin, n (%)	57 (10.05)	44 (20.28)	<0.001	33 (11.91)	34 (18.28)	0.056
	ICD, n (%)	13 (2.29)	12 (5.53)	0.021	11 (3.97)	8 (4.30)	0.861
	CRT, n (%)	57 (10.05)	5 (2.30)	<0.001	12 (4.33)	5 (2.69)	0.356

Data presented as frequencies (percentage) for categorical variables and median (IQR) for continuous variables. AF, atrial fibrillation; ACEI/ARB/ARNI, angiotensin-converting enzyme inhibitors/angiotensin II receptor antagonists/angiotensin receptor–neprilysin inhibitors; AR, aortic regurgitation; AVB, atrioventricular block; BMI, body mass index; CKD, chronic kidney disease; CRT, cardiac resynchronization therapy; DBP, diastolic blood pressure; DM, diabetes mellitus; eGFR, estimated glomerular filtration rate; fQRS, fragmented QRS complex; ICD, implantable cardioverter-defibrillator; IQR, interquartile range; IVB, intraventricular block; LAFB, left anterior fascicular block; LBBB, left bundle branch block; Log NT-proBNP, log-transformed N-terminal pro-B-type natriuretic peptide; LPFB, left posterior fascicular block; LQRSV, low QRS voltages; LVEDDI, left ventricular end-diastolic diameter index; LVEF, left ventricular ejection fraction; MR, mitral regurgitation; MRA, mineralocorticoid receptor antagonists; NIDCM, non-ischemic dilated cardiomyopathy; NSVT, non-sustained ventricular tachycardia; NYHA, New York Heart Association; PSM, propensity score matching; SBP, systolic blood pressure; Scr, serum creatinine; SGLT2i, sodium-glucose cotransporter-2 inhibitor; TR, tricuspid regurgitation.

### 3.2 Primary and Secondary Outcomes

During a median follow-up of 1079 days, 335 primary outcomes occurred in the overall cohort, with 171 events in the LQRSV group and 164 events in the non-LQRSV group (78.80% vs. 28.92%, *p* < 0.001). In the matched cohort, the incidence of the primary outcome was significantly higher in the LQRSV group than in the non-LQRSV group (77.42% vs. 36.46%, *p* < 0.001). Incidents of all-cause mortality (50.00% vs. 16.25%, *p* < 0.001), CV death (46.77% vs. 13.00%, *p* < 0.001), and HF re-hospitalization (45.70% vs. 24.55%, *p* < 0.001) were also significantly higher in the LQRSV group (Table 2).

**Table 2.** **Incidence of primary and secondary outcomes before and after PSM in patients with NIDCM**.

Outcomes		Before PSM			After PSM		
		Non-LQRSV (n = 567)	LQRSV (n = 217)	*p*-value	Non-LQRSV (n = 277)	LQRSV (n = 186)	*p*-value
Primary outcome, n (%)		164 (28.92)	171 (78.80)	<0.001	101 (36.46)	144 (77.42)	<0.001
Secondary outcomes							
	All-cause mortality, n (%)	69 (12.17)	116 (53.46)	<0.001	45 (16.25)	93 (50.00)	<0.001
	CV death, n (%)	54 (9.52)	107 (49.31)	<0.001	36 (13.00)	87 (46.77)	<0.001
	HF re-hospitalization, n (%)	114 (20.11)	91 (41.94)	<0.001	68 (24.55)	85 (45.70)	<0.001

The primary outcome was the composite of all-cause mortality or HF re-hospitalization. CV death, cardiovascular death; HF, heart failure; LQRSV, low QRS voltage; PSM, propensity score matching.

Kaplan–Meier survival analysis demonstrated that patients in the LQRSV group had a significantly higher proportion of the composite of all-cause mortality or HF re-hospitalization than those in the non-LQRSV group (*p* < 0.001). Rates of all-cause mortality, CV death, and HF re-hospitalization were also significantly higher in the LQRSV group (Fig. 2).

**Fig. 2.**
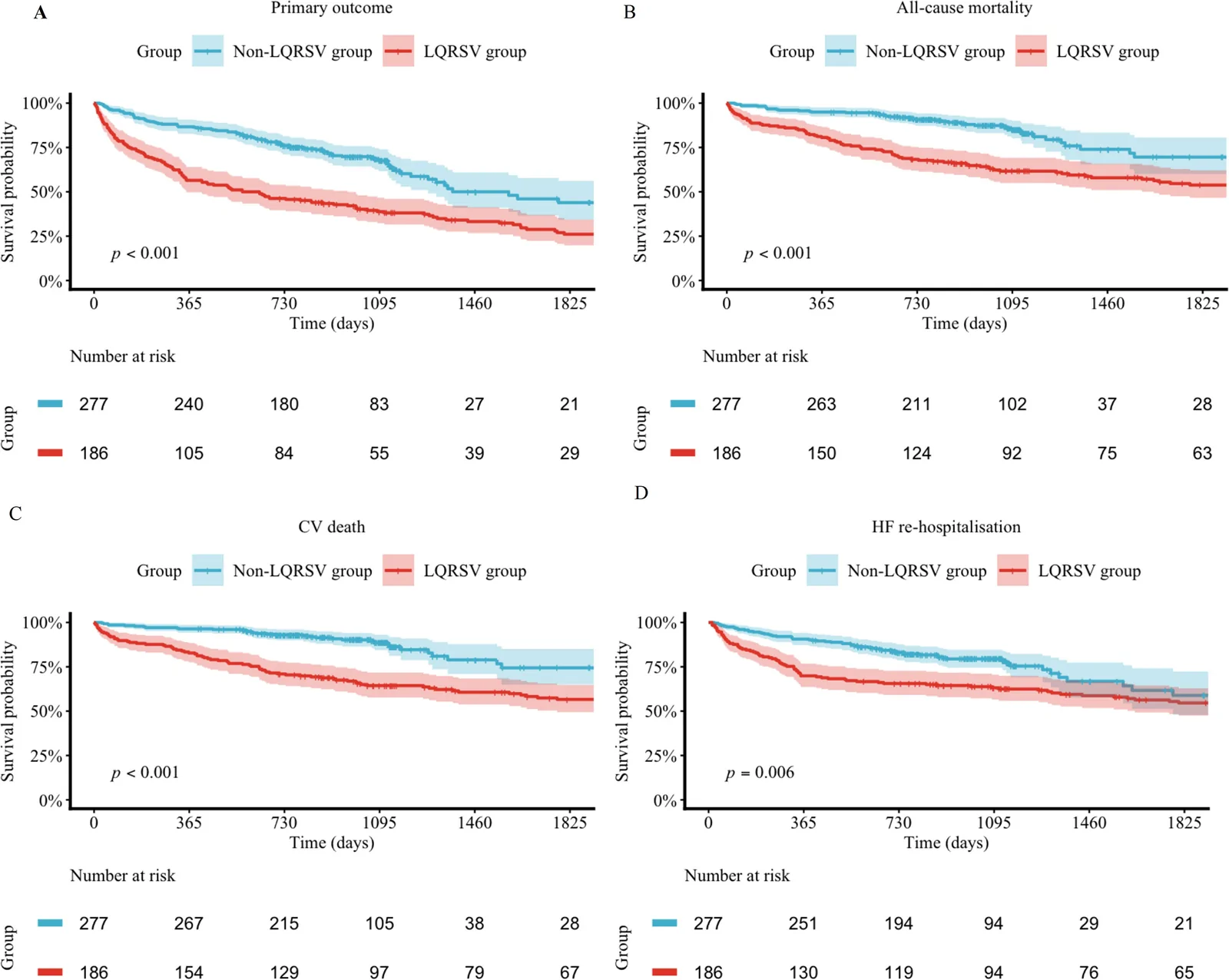
**Kaplan‒Meier curves in the matched cohort**. (A) Primary outcome. (B) All-cause mortality. (C) Cardiovascular death. (D) Heart failure re-hospitalization. LQRSV, low QRS voltage; CV, cardiovascular.

In univariate Cox regression analysis for the primary outcome, covariates including LQRSV, age, BMI, NYHA class III/IV, CKD, QRS duration, AF, LVEDDI, LVEF, log NT-proBNP, moderate-to-severe MR, moderate-to-severe TR and SGLT2i use were significantly associated with the composite of all-cause mortality or HF re-hospitalization (Table 3). Multivariate analysis revealed that LQRSV was an independent risk factor for predicting the composite outcome of all-cause mortality or HF re-hospitalization (HR 2.048, 95% CI 1.519‒2.760; *p* < 0.001). Additionally, LQRSV was also independently and closely related to all-cause mortality (HR 2.526, 95% CI 1.660–3.844; *p* < 0.001) and CV death (HR 2.820, 95% CI 1.776‒4.476; *p* < 0.001) (**Supplementary Table 2**). Subgroup analysis revealed that LQRSV was an independent risk factor for the primary outcome (Fig. 3).

**Table 3.** **Factors associated with primary outcome in the matched cohort**.

	Univariate analysis		Multivariate analysis	
	HR (95% CI)	*p*-value	HR (95% CI)	*p*-value
LQRSV	2.249 (1.735–2.917)	<0.001	2.048 (1.519–2.760)	<0.001
Age	1.011 (1.001–1.021)	0.027		
BMI	0.957 (0.928–0.987)	0.005		
NYHA class III/IV	2.201 (1.566–3.093)	<0.001	1.624 (1.121–2.351)	0.010
CKD	1.476 (1.084–2.01)	0.013		
QRS duration	1.009 (1.004–1.013)	<0.001	1.007 (1.002–1.011)	0.002
AF	1.474 (1.122–1.937)	0.005		
LVEDDI	1.018 (1.009–1.027)	<0.001		
LVEF	0.987 (0.975–0.999)	0.038		
Log NT-proBNP	2.154 (1.719–2.698)	<0.001	2.089 (1.606–2.717)	<0.001
SGLT2i	0.482 (0.365–0.637)	<0.001		
MR (moderate/severe)	1.515 (1.152–1.994)	0.003		
TR (moderate/severe)	1.534 (1.191–1.975)	<0.001		

The Cox proportional hazards model was adjusted for LQRSV, NYHA III/IV class, QRS duration, AF, LVEF, Log NT-proBNP, and SGLT2i. AF, atrial fibrillation; BMI, body mass index; CKD, chronic kidney disease; Log NT-proBNP, log-transformed N-terminal pro-B-type natriuretic peptide; LQRSV, low QRS voltage; LVEDDI, left ventricular end-diastolic diameter index; LVEF, left ventricular ejection fraction; MR, mitral regurgitation; NYHA, New York Heart Association; SGLT2i, sodium-glucose cotransporter 2 inhibitor; TR, tricuspid regurgitation.

**Fig. 3.**
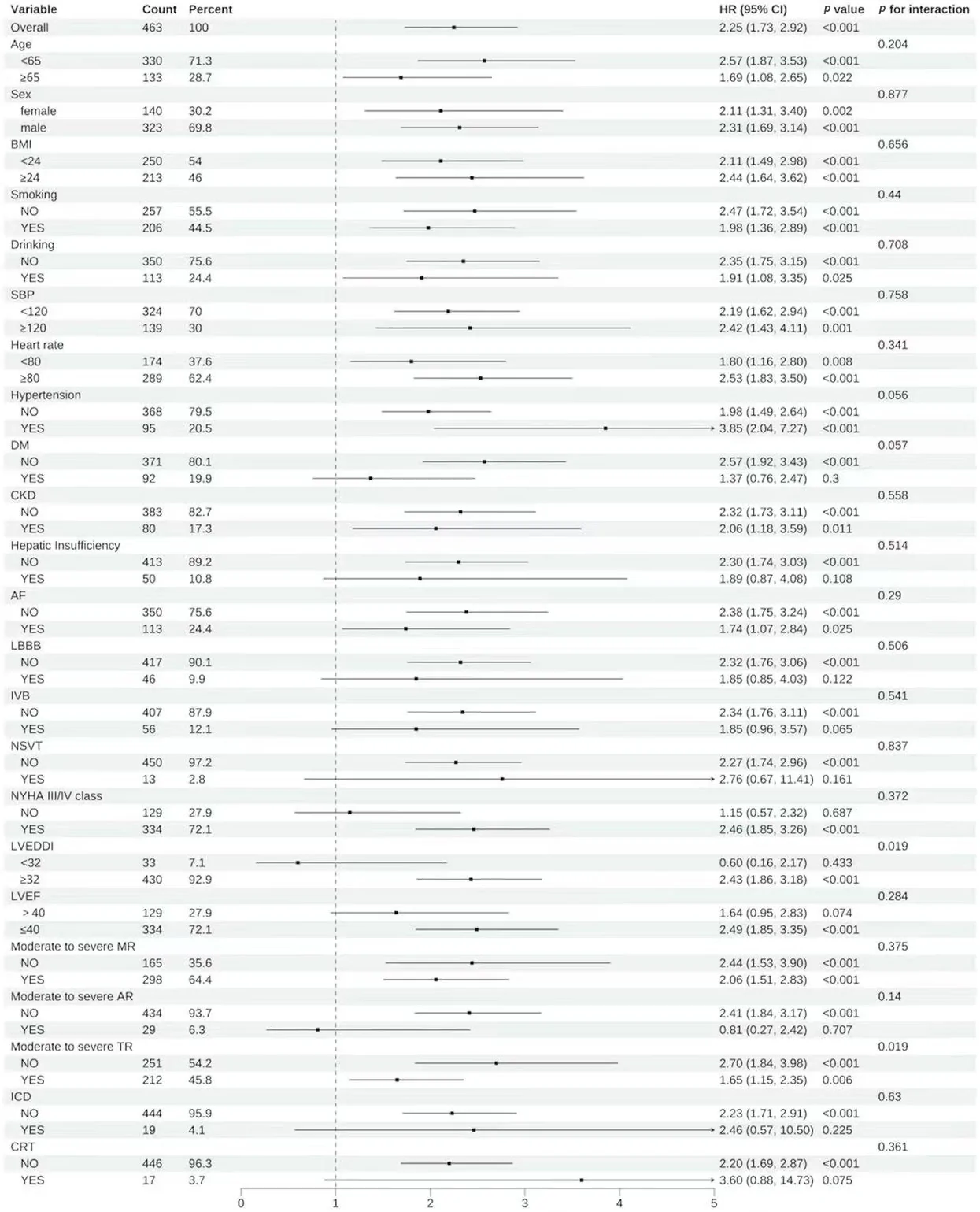
**Subgroup analysis for the primary outcome in the matched cohort**. AF, atrial fibrillation; AR, aortic regurgitation; BMI, body mass index; CKD, chronic kidney disease; CRT, cardiac resynchronization therapy; DM, diabetes mellitus; ICD, implantable cardioverter-defibrillator; LBBB, left bundle branch block; LVEDDI, left ventricular end-diastolic diameter index; LVEF, left ventricular ejection fraction; MR, mitral regurgitation; NSVT, non-sustained ventricular tachycardia; NYHA, New York Heart Association; SBP, systolic blood pressure.

### 3.3 QRS Voltages and Outcomes

In the matched cohort, the LQRSV group showed significantly lower minimum limb-lead voltage (0.11 vs. 0.40 mV, *p* < 0.001), limb-lead voltage sum (1.40 vs. 4.04 mV, *p* < 0.001), and SV1 + RV5 voltage (1.61 vs. 2.76 mV, *p* < 0.001) compared with the non-LQRSV group (Table 1). RCS analysis was used to evaluate the relationships between QRS voltage parameters and the primary outcome. As shown in Fig. 4, for all three voltage indices (minimum limb-lead voltage, voltage sum in limb leads, and SV1 + RV5 voltage), HR decreased continuously as voltage increased. The highest risk was observed at lower voltage levels, followed by a rapid decline toward an HR close to 1.0, stabilizing thereafter. For each parameter, the overall test for association was statistically significant (*p* < 0.001), indicating that voltage levels were independently associated with the outcome. However, nonlinear components were not statistically significant (nonlinear, *p* > 0.05), suggesting that the relationship was predominantly linear rather than curvilinear across the voltage range. These findings demonstrate that lower QRS voltage is continuously and linearly associated with a higher risk of adverse events, reflecting a dose-response pattern.

**Fig. 4.**
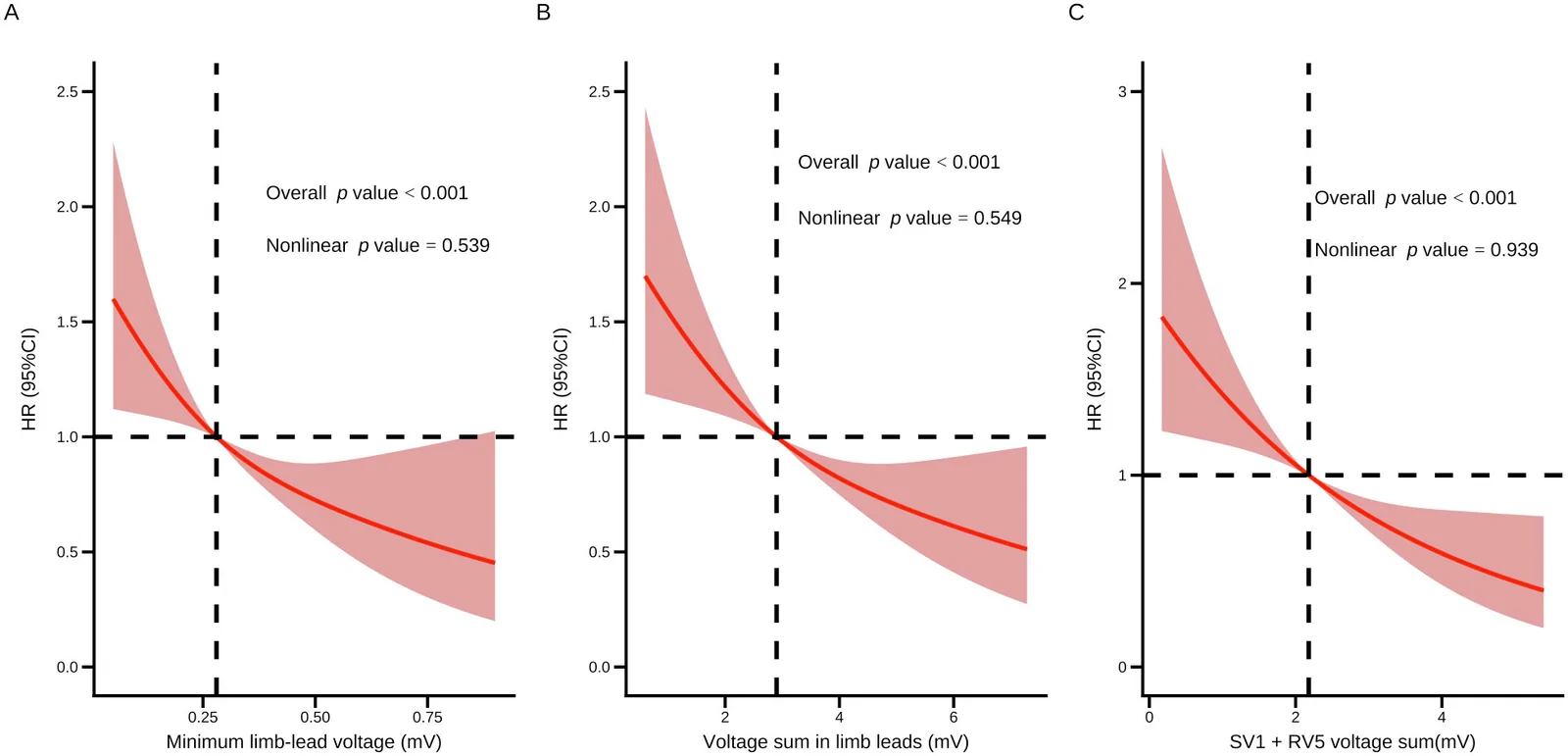
**Restricted cubic spline models illustrate the dose–response relationships between three QRS voltage parameters and the primary outcome in the matched cohort**. (A) Minimum limb-lead voltage. (B) Voltage sum in limb leads. (C) SV1+RV5 voltage sum.

### 3.4 ROC Curves

ROC curves were used to evaluate the predictive value of LQRSV. As shown in Fig. 5A, Model 1 (including LQRSV, NYHA class, log NT-proBNP, and QRS duration) showed better prognostic performance than Model 2 (without LQRSV) (AUC 0.808 vs. 0.723, *p* < 0.001). The predictive performance of individual voltage indices—minimum limb-lead voltage, voltage sum, and SV1 + RV5 voltage—showed AUC of 0.682, 0.69, and 0.696, respectively (Fig. 5B), indicating modest but consistent discriminatory ability.

**Fig. 5.**
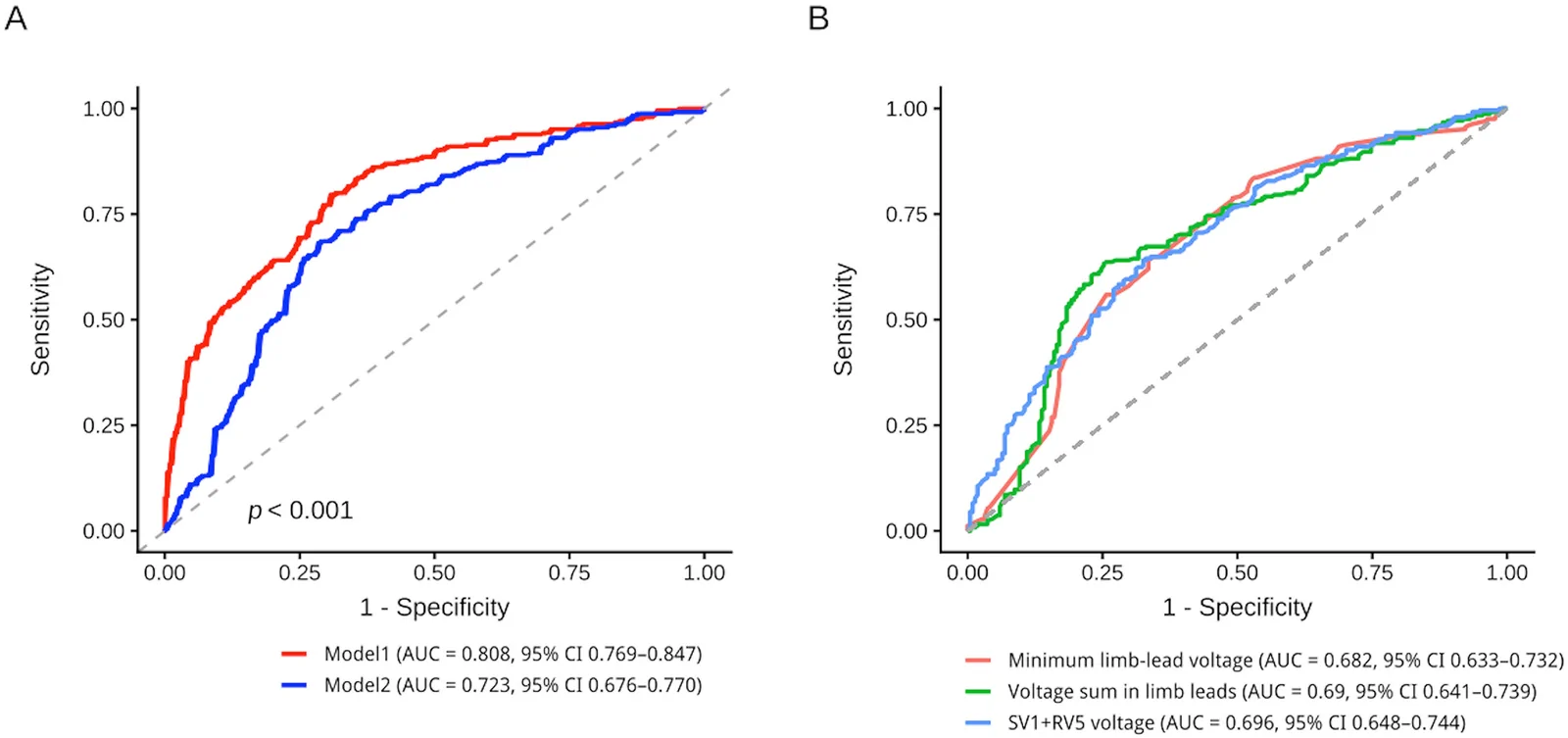
**ROC curve analyses for the primary outcome in the matched cohort**. (A) ROC curve analyses of LQRSV. Model 1 included LQRSV, NYHA class III/IV, QRS duration, and log NT-proBNP; Model 2 included NYHA class III/IV, QRS duration, and log NT-proBNP. (B) ROC curve analyses of QRS voltages for composite outcome. AUC, area under the curve.

### 3.5 Sensitivity Analysis

In the overall cohort, Kaplan–Meier curves revealed that patients in the LQRSV group had a significantly higher proportion of composite all-cause mortality or HF re-hospitalization, all-cause mortality, CV death, and HF re-hospitalization compared with those in the non-LQRSV group (**Supplementary Fig. 1**). Univariate Cox regression analyses demonstrated that LQRSV remained significantly associated with the primary and three secondary outcomes. Following multivariable adjustment, the association between LQRSV and HF re-hospitalization lost statistical significance. In contrast, associations with other outcome events (composite of all-cause mortality or HF re-hospitalization, all-cause mortality, and CV death) remained significant (**Supplementary Table 2**), consistent with findings from the matched cohort.

## 4. Discussion

In this study, we investigated the prognostic implications of LQRSV in a cohort of patients with NIDCM. We observed that the presence of LQRSV was a significant and independent predictor of the composite of all-cause mortality or HF re-hospitalization, all-cause mortality, and CV death in patients with NIDCM, after adjusting for traditional risk factors such as NYHA functional class, QRS duration, and log NT-proBNP. These findings were consistent across both subgroup and sensitivity analyses, further confirming the robustness of our results. Moreover, the risk associated with LQRSV was closely related to voltage amplitude.

RCS analysis revealed a predominantly linear association between QRS voltage and adverse outcomes, with no significant evidence of nonlinearity. This finding suggests that the prognostic value of QRS voltage is not limited to a simple present-or-absent threshold effect based on the conventional definition of LQRSV. Instead, the risk increased continuously as QRS voltage decreased. Therefore, even borderline lower QRS voltage may indicate higher risk than relatively preserved voltage, supporting the clinical value of QRS voltage assessment as a continuous marker in addition to binary LQRSV classification.

An important nuance was observed for HF re-hospitalization. Although patients with LQRSV had a higher incidence of HF re-hospitalization and worse event-free survival, the association between LQRSV and HF re-hospitalization alone was attenuated after multivariable adjustment. HF re-hospitalization is a multifactorial endpoint influenced by baseline disease severity, treatment patterns, physician decision-making, healthcare access, and follow-up intensity. In addition, death may act as a competing risk for HF re-hospitalization, particularly in patients with advanced disease, because patients who die early may no longer experience subsequent HF re-hospitalization. This competing-risk effect, together with the retrospective nature of the study and potential residual confounding, may partly explain the less robust independent association between LQRSV and HF re-hospitalization alone. Therefore, the prognostic association of LQRSV appeared more consistent for the composite outcome and mortality-related outcomes than for HF re-hospitalization alone.

LQRSV was initially defined as the sum of the S-wave amplitude in lead V1 and the R-wave amplitude in leads V5 or V6 <15 mm (1.5 mV) and subsequently simplified to a nadir-to-peak QRS amplitude <5 mm in all limb leads and <10 mm in all precordial leads [8,26]. It is attributed to conditions that impair either the initiation of cardiac electrical impulses or their conduction to surface recording electrodes [21]. In patients with NIDCM, LQRSV occurs in 6‒7% of cases but may be higher (36%) in certain genetic forms [27]. In NIDCM, QRS voltage may vary according to disease stage. In earlier or relatively compensated stages, increased ventricular mass, ventricular hypertrophy, or chamber enlargement may be associated with preserved or even increased QRS voltage. However, with disease progression, extensive myocardial fibrosis, replacement scar formation, loss of viable electrically active myocardium, and worsening cardiac function may reduce surface QRS voltage. Therefore, the presence of LQRSV in patients with NIDCM may indicate advanced myocardial remodeling and functional deterioration rather than simply reflecting ventricular size [8,14,21]. This may explain why patients with LQRSV had more severe clinical features and worse long-term outcomes in the present study. The underlying pathological mechanisms can be categorized as follows. First, extensive myocardial fibrosis, one of the core pathological features of NIDCM, is strongly associated with adverse ventricular remodeling and increased susceptibility to malignant ventricular arrhythmias [28]. Fibrous scar tissue lacks electrical conductivity and physically blocks the conduction pathways between cardiomyocytes, diminishing the amplitude of surface QRS complexes [6]. Second, significant myocardial cell loss directly weakens the intensity of myocardial depolarization, presenting as LQRSV [29]. Concurrently, massive cardiomyocyte loss leads to progressive decline in LV systolic function, triggering severe HF and increasing the risk of readmission and all-cause mortality. Third, advanced NIDCM is frequently accompanied by severe right HF or biventricular failure, resulting in systemic congestion and peripheral edema. Although clinically relevant pericardial or pleural effusion was excluded in this study, systemic congestion and tissue edema in advanced HF may theoretically contribute to attenuation of surface QRS voltage [21].

In particular, Calò et al. [30] reported that reduced limb lead and precordial/lateral voltage indices in arrhythmogenic left ventricular cardiomyopathy were associated with myocardial fibrosis and arrhythmogenic substrates. Similarly, the lower limb lead voltage sum and lower combined voltage of the S wave in V1 and the R wave in V5 observed in our LQRSV group may reflect advanced myocardial remodeling and loss of electrically active myocardium. Therefore, LQRSV is not merely an electrical phenomenon; it is a composite manifestation of severe myocardial matrix remodeling and end-stage pathophysiological deterioration in patients with NIDCM. Consistent with our findings, patients with LQRSV demonstrated worse NYHA classification, higher NT-proBNP levels, and a greater number of comorbidities. Additionally, LQRSV signals exhaustion of contractile reserve and an increased risk of lethal arrhythmias, making it a robust independent risk factor for all-cause mortality and re-hospitalization. Low voltage in NIDCM is independently associated with late gadolinium enhancement (LGE) on cardiac magnetic resonance imaging and has prognostic value for predicting sudden cardiac death, sustained ventricular arrhythmias, or appropriate ICD therapy [5,31]. Therefore, LQRSV can occur in NIDCM and is linked to adverse outcomes.

Our results are consistent with those of previous studies. Li et al. [14] reported that in a large population with CV diseases, LQRSV was associated with severe cardiac dysfunction and adverse clinical outcomes. Beyond this observation, the poor prognostic relevance of LQRSV has been consistently demonstrated across different cardiac conditions, including cardiac amyloidosis, hypertrophic cardiomyopathy, and arrhythmogenic right ventricular cardiomyopathy [11,12,15]. In addition, recent literature has emphasized the role of ECG-derived and electrophysiological markers in risk stratification across the non-ischemic cardiomyopathy spectrum. Milaras et al. [32] reported that electrophysiological parameters, including QRS duration, QTc interval, late potentials, premature ventricular complex burden, and nonsustained ventricular tachycardia, were associated with phenotypic differences and adverse events in patients with non-ischemic cardiomyopathy. These findings support the broader concept that ECG-based markers may provide prognostic information beyond LVEF alone. In the present study, lower QRS voltages were associated with a higher risk of all-cause mortality or HF re-hospitalization, suggesting a more advanced clinical stage and worse prognosis. Collectively, these data reinforce the concept that LQRSV is not merely a benign ECG finding but a reflection of advanced myocardial disease.

## 5. Limitations

Nevertheless, this study had some limitations. First, as a retrospective, single-center study, the generalizability of the results is limited. Second, cardiac magnetic resonance imaging was not systematically available. Thus, we could not assess the relationship between LQRSV and myocardial fibrosis or scar burden. Third, genetic testing was not routinely performed. Therefore, we could not evaluate the influence of high-risk variants (*e.g.*, *DSP*, *LMNA*, *FLNC*, *RBM20*), which may contribute to residual confounding. Fourth, although we excluded other diseases known to cause LQRSV, such as COPD and clinically relevant pericardial disease, ECG voltage may still be affected by obesity, cardiac amyloidosis, and other unmeasured extracardiac factors, which may confound the association. Finally, the absence of repeated ECG and Holter monitoring data limited our ability to assess dynamic changes in LQRSV and arrhythmic burden during follow-up. However, the use of a single baseline ECG remains clinically practical for routine risk assessment. Future prospective studies integrating serial ECG assessment, cardiac imaging, genetic testing, and biomarker data are warranted to elucidate the pathophysiological basis and prognostic utility of LQRSV in patients with NIDCM.

## 6. Conclusions

Our findings indicate that ECG-detected LQRSV is independently associated with worse clinical outcomes in patients with NIDCM. As a readily accessible and cost-effective tool, LQRSV may serve as a valuable adjunct in the risk stratification of NIDCM, aiding clinicians in identifying patients with advanced disease and guiding individualized management strategies.

## Data Availability

The datasets generated or analyzed for the present study are available from the corresponding authors upon reasonable request, in accordance with institutional data sharing policies.

## Abbreviations

ACEI/ARB/ARNI, angiotensin-converting enzyme inhibitors/angiotensin II receptor antagonists/angiotensin receptor–neprilysin inhibitors; AF, atrial fibrillation; AR, aortic regurgitation; AUC, area under the curve; AVB, atrioventricular block; BMI, body mass index; CI, confidence interval; CKD, chronic kidney disease; COPD, chronic obstructive pulmonary disease; CRT, cardiac resynchronization therapy; CV, cardiovascular; DBP, diastolic blood pressure; DM, diabetes mellitus; eGFR, estimated glomerular filtration rate; fQRS, fragmented QRS complex; HF, heart failure; HR, hazard ratios; ICD, implantable cardioverter-defibrillator; IQR, interquartile range; IVB, intraventricular block; LAFB, left anterior fascicular block; LBBB, left bundle branch block; Log NT-proBNP, log-transformed N-terminal pro-B-type natriuretic peptide; LPFB, left posterior fascicular block; LQRSV, low QRS voltages; LV, left ventricular; LVEDDI, left ventricular end-diastolic diameter index; LVEF, left ventricular ejection fraction; MR, mitral regurgitation; MRA, mineralocorticoid receptor antagonists; NIDCM, non-ischemic dilated cardiomyopathy; NSVT, non-sustained ventricular tachycardia; NYHA, New York Heart Association; PSM, propensity score matching; RCS, restricted cubic spline; ROC, receiver operating characteristic; Scr, serum creatinine; SBP, systolic blood pressure; SGLT2i, sodium-glucose cotransporter 2 inhibitor; TR, tricuspid regurgitation; VIF, variance inflation factor.
