# Supplementary material for: Clinical Correlates and Prognostic Value of Low QRS Voltages in Non-Ischemic Dilated Cardiomyopathy: A Retrospective Cohort Study
Source: Rev Cardiovasc Med. 2026 Oct 22;27(10):50413. doi: 10.31083/RCM50413 (PMC13732673; doi:10.31083/RCM50413)
Supplement: Supplementary file 1 [file 2153-8174-27-10-50413-s1.zip › Supplementary Material.docx]

Supplementary Material

**Supplementary Fig. 1. Kaplan‒Meier curve in the overall cohort.** CV, cardiovascular; HF, heart failure.

**Supplementary Table 1. Baseline characteristics before and after PSM.**

|  | Before PSM | |  | After PSM | | | |  |
| --- | --- | --- | --- | --- | --- | --- | --- | --- |
|  | Non-LQRSV  (n=567) | LQRSV  (n=217) | SMD | Non-LQRSV  (n=277) | LQRSV  (n=186) | SMD | |  |
| Age, year, median (IQR) | 57 (48, 64) | 54 (44, 67) | -0.071 | 57 (49, 66) | 56 (45, 68) | -0.044 |  |  |
| Male, n (%) | 400 (70.55) | 149 (68.66) | -0.019 | 193 (69.68) | 130 (69.89) | 0.008 |  |  |
| BMI, kg/m^2^, median (IQR) | 24.2 (21.5, 27.0) | 23.3 (20.4, 26.0) | -0.191 | 23.6 (21.1, 27.0) | 23.3 (20.5, 26.4) | -0.018 |  |  |
| Smoking, n (%) | 247 (43.56) | 98 (45.16) | 0.016 | 124 (44.77) | 82 (44.09) | 0.005 |  |  |
| Drinking, n (%) | 153 (26.98) | 49 (22.58) | -0.044 | 67 (24.19) | 46 (24.73) | 0.019 |  |  |
| NYHA class III/IV, % | 272 (47.97) | 173 (79.72) | 0.318 | 192 (69.31) | 142 (76.34) | 0.000 |  |  |
| SBP, mmHg, median (IQR) | 113 (100, 126) | 103 (91, 117) | -0.396 | 107 (95, 122) | 106 (95, 120) | 0.038 |  |  |
| Hypertension, n (%) | 155 (27.34) | 38(17.51) | -0.098 | 59 (21.30) | 36 (19.35) | 0.000 |  |  |
| CKD, n (%) | 65 (11.46) | 46 (21.20) | 0.097 | 47 (16.97) | 33 (17.74) | -0.013 |  |  |
| Hepatic insufficiency, n (%) | 32 (5.64) | 33 (15.21) | 0.096 | 27 (9.75) | 23 (12.37) | 0.013 |  |  |
| QRS duration, ms, median (IQR) | 104 (90, 132) | 98 (86, 116) | -0.287 | 100 (86, 124) | 98 (86, 118) | -0.046 |  |  |
| AF, n (%) | 86 (15.17) | 62 (28.57) | 0.134 | 60 (21.66) | 53 (28.49) | 0.027 |  |  |
| LBBB, n (%) | 108 (19.05) | 17 (7.83) | -0.112 | 29 (10.47) | 17 (9.14) | 0.008 |  |  |
| LVEDDI, mm/m^2^, median (IQR) | 45 (38, 54) | 47 (40, 56) | 0.137 | 46 (39, 55) | 47 (40, 56) | 0.002 |  |  |
| LVEF, median (IQR) | 36 (28, 44) | 35 (29, 42) | -0.133 | 34 (28, 41) | 35 (29, 42) | 0.008 |  |  |
| log NT-proBNP, median (IQR) | 3.12 (2.56, 3.61) | 3.51 (3.21, 3.97) | 0.687 | 3.37 (2.83, 3.86) | 3.44 (3.18, 3.83) | -0.023 |  |  |
| ICD, n (%) | 13 (2.3) | 12 (5.5) | 0.032 | 11 (4.0) | 8 (4.3) | 0.003 |  |  |
| CRT, n (%) | 57 (10) | 5 (2.3) | -0.078 | 12 (4.3) | 5 (2.7) | -0.008 |  |  |

AF, atrial fibrillation; BMI, body mass index; CKD, chronic kidney disease; CRT, cardiac resynchronization therapy; ICD, implantable cardioverter-defibrillator; LBBB, left bundle branch block; Log NT-proBNP, log-transformed N-terminal pro-B-type natriuretic peptide; LQRSV, low QRS voltage; LVEDDI, left ventricular end-diastolic diameter index; LVEF, left ventricle ejection fraction; NIDCM, non-ischemic dilated cardiomyopathy; NYHA, New York Heart Association; PSM, propensity score matching.

**Supplementary Table 2. Association between LQRSV and outcome events before and after PSM.**

| Outcomes | Univariate analysis | | Multivariate analysis | |
| --- | --- | --- | --- | --- |
|  | HR (95% CI) | *p* | HR (95% CI) | *p* |
| After PSM |  |  |  |  |
| All-cause mortality | 2.592 (1.798–3.736) | < 0.001 | 2.526 (1.660–3.844) | < 0.001 |
| CV death | 2.959 (1.986–4.408) | < 0.001 | 2.820 (1.776–4.476) | < 0.001 |
| HF re-hospitalization | 1.588 (1.139–2.215) | 0.006 | 1.222 (0.835–1.787) | 0.302 |
| Before PSM |  |  |  |  |
| Primary outcome | 3.173 (2.548–3.95) | < 0.001 | 2.112 (1.642–2.715) | < 0.001 |
| All-cause mortality | 3.923 (2.886–5.332) | < 0.001 | 2.509 (1.769–3.558) | < 0.001 |
| CV death | 4.459 (3.183–6.245) | < 0.001 | 2.634 (1.794–3.868) | < 0.001 |
| HF re-hospitalization | 2.302 (1.600-3.313) | < 0.001 | 1.120 (0.793-1.583) | 0.519 |

CV, cardiovascular; HF, heart failure; LQRSV, low QRS voltage; PSM, propensity score matching
